# Supplementary material for: Development of High Affinity Calcitonin Analog Fragments Targeting Extracellular Domains of Calcitonin Family Receptors
Source: Biomolecules. 2021 Sep 15;11(9):1364. doi: 10.3390/biom11091364 (PMC8466238; doi:10.3390/biom11091364)
Supplement: Supplementary file 1 [file biomolecules-11-01364-s001.zip › Sup. info_Biomolecules_Lee_091421_Proofreading_TC_accepted.pdf]

Supplemental information

Title: Development of high affinity calcitonin analog fragments targeting extracellular domains of calcitonin family receptors

Author: Sangmin Lee*

Department of Basic Pharmaceutical Sciences,
Fred Wilson School of Pharmacy, High Point University, High Point, NC, 27268 USA

*Corresponding author
Sangmin Lee, Ph.D.
Email: slee2@highpoint.edu

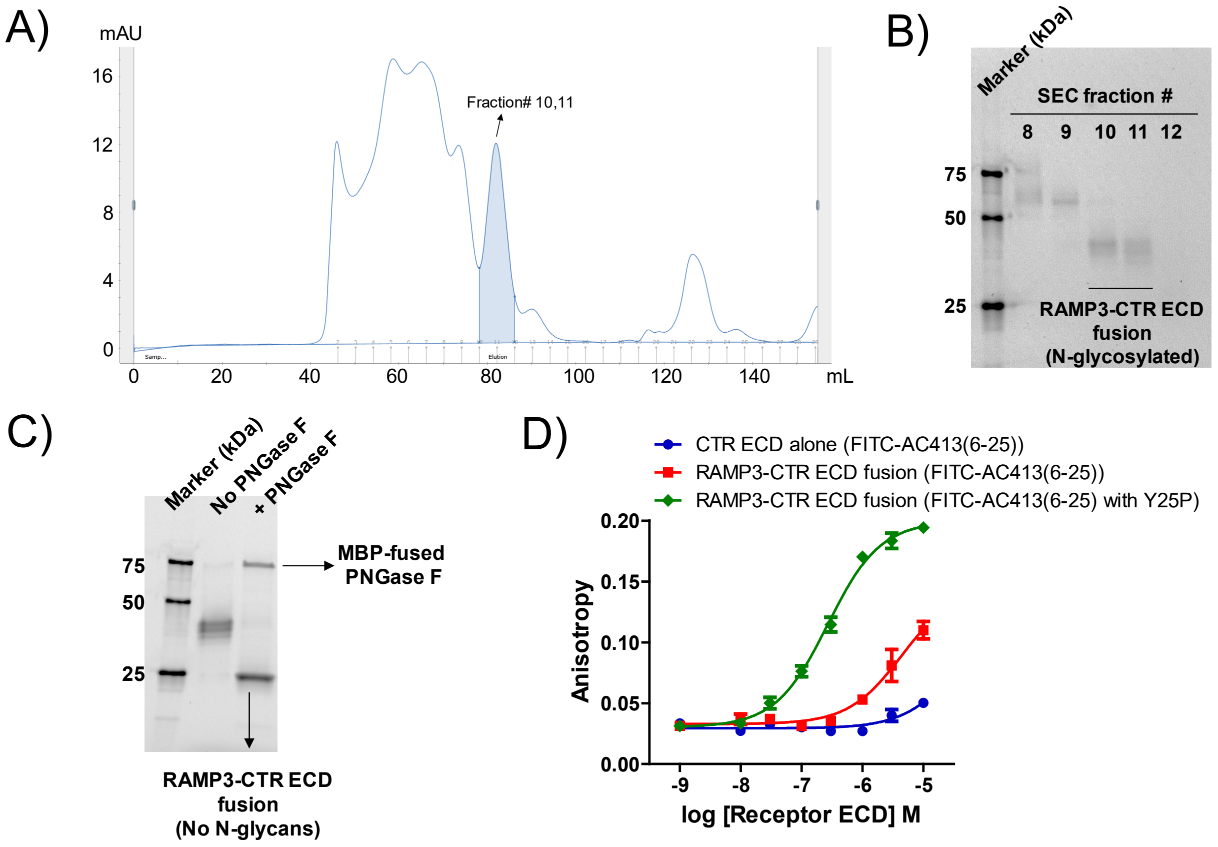

Figure S1. Purification of the RAMP3-CTR ECD fusion protein and its selective binding for an antagonistic amylin analog AC413. A) Size exclusion column (SEC) chromatography elution profile. The peak fractions (fraction number 10 and 11) were collected and dialyzed to storage buffer. B) SDS-PAGE with the fractions from SEC chromatography showed the purified and N-glycosylated RAMP3-CTR ECD fusion protein. C) PNGase F treatment with the purified and N-glycosylated RAMP3-CTR ECD fusion protein. The RAMP3-CTR ECD fusion protein was incubated with maltose-binding protein (MBP)-tagged PNGase F at 1:10 ratio (w/w, PNGase F: RAMP3-CTR ECD fusion protein) with the reaction buffer (50 mM Sodium Phosphate, pH 7.5) for 5h at 37˚C. The RAMP3-CTR ECD fusion protein without N-glycans showed a protein band close to 25 kDa, the expected MW. D) FP peptide binding assay with CTR ECD alone and the RAMP3-CTR ECD fusion protein. Either FITC-labeled AC413(6–25) WT or with Y25P mutation was used as a peptide probe. FITC-labeled AC413(6–25) showed selective binding for the RAMP3-CTR ECD fusion protein over CTR ECD alone. FITC-labeled AC413(6–25) with Y25P mutation showed a great affinity increase for the RAMP3-CTR ECD fusion protein compared to FITC-labeled AC413(6–25) and it was used as a peptide probe for FP peptide binding assay with AMY receptor ECDs due to its high affinity.

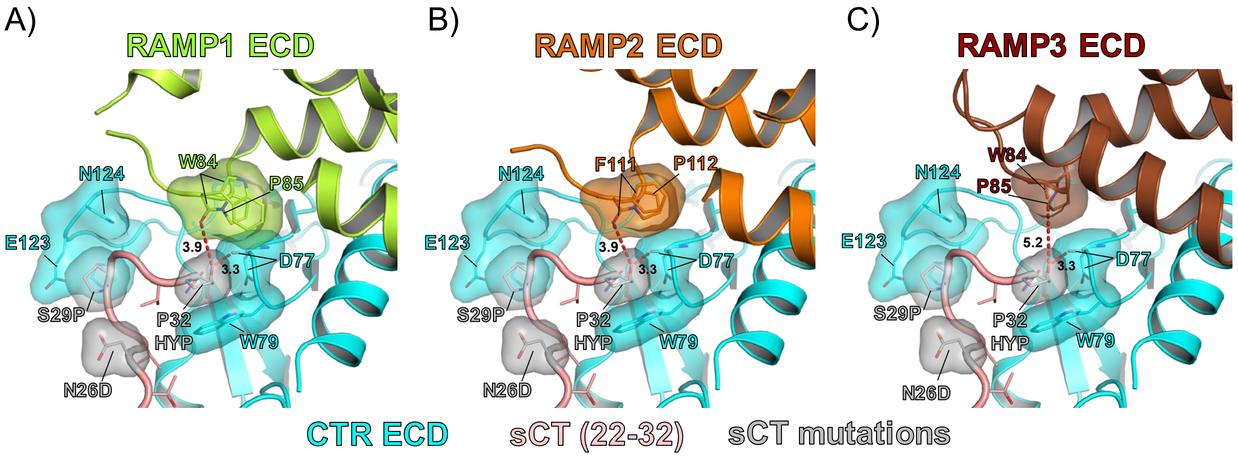

Figure S2. Additional hypothetical structures of the RAMP-CTR ECD fusion proteins and sCT(22–32) with N26D/S29P/P32HYP mutations. A and B) Crystal structures of the RAMP1-CLR ECD fusion protein (PDB 4RWG), the RAMP2-CLR ECD fusion protein (PDB 4RWF), and CTR ECD with an sCT fragment (PDB 6PFO, Mol A) were used to build the hypothetical structures of RAMP1-CTR ECD fusion and RAMP2-CTR ECD fusion proteins. C) The cryo-EM structure of the AM2 receptor (PDB 6UUS) with AM peptide bound and the crystal structure of CTR ECD with an sCT fragment (PDB 6PFO, Mol A) were used to build the hypothetical structure of the RAMP3-CTR ECD fusion protein. CTR ECD D77, W79, H121, E123 and N124, sCT N26D, S29P, and P32HYP mutated residues, and RAMP ECD residues (W84 and P85 in RAMP1/3 ECD and F111 and P112 in RAMP2 ECD) were shown both with stick and surface representations. For the RAMP3-CTR ECD fusion protein, the side chain of W84 of RAMP3 ECD was unavailable in the original cryo-EM structure (PDB 6UUS) and only the first carbon of the side chain was shown as stick and surface representations. The distances between sCT HYP32 and the proximal RAMP ECD residues and between sCT HYP32 and the CTR ECD D77 main chain were shown as a dotted red line and the measurement (Å) was indicated in black.

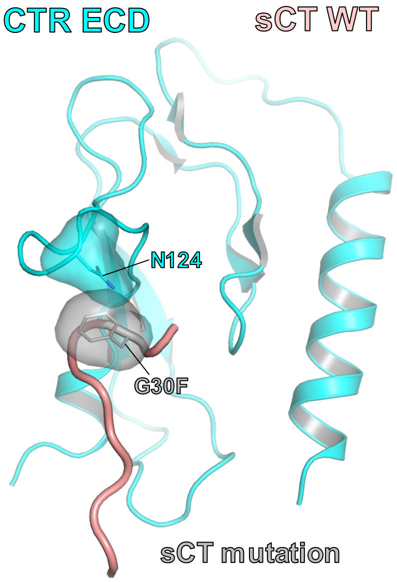

Figure S3. The hypothetical structure of sCT(22–32) with G30F mutation at CTR ECD. The crystal structure of CTR ECD (PDB 6PFO, Mol A) with an sCT fragment was used to build the hypothetical structure. sCT(22–32) G30F mutation was made using mutagenesis function in Pymol. CTR ECD N124 and sCT G30F mutated residue were shown both with stick and surface representations.
